# Healthcare providers' perspectives and needs related to the management of Pediatric Feeding Disorder: A focus group study

**DOI:** 10.1371/journal.pone.0354724

**Published:** 2026-07-29

**Authors:** Kelsey Thompson, Cuyler Romeo, Meg Simione, Hayley Estrem, Jaclyn L. Pederson, William G. Sharp

**Affiliations:** 1 Division of Speech and Hearing Sciences, University of North Carolina at Chapel Hill, Chapel Hill, North Carolina, United States of America; 2 Feeding Matters, Phoenix, Arizona, United States of America; 3 Banner University Medical Center – Tucson, Tucson, Arizona, United States of America; 4 Department of Communicative Disorders, University of Rhode Island, Kingston, Rhode Island, United States of America; 5 School of Nursing, University of North Carolina at Wilmington, Wilmington, North Carolina, United States of America; 6 Department of Pediatrics, Emory University School of Medicine, Atlanta, Georgia, United States of America; 7 Multidisciplinary Feeding Program, Children’s Healthcare of Atlanta, Atlanta, Georgia, United States of America; Manipal Academy of Higher Education, INDIA

## Abstract

Pediatric feeding disorder is a prevalent, impactful diagnosis for children and their families. This diagnosis is heterogenous in presentation and requires the care of a multidisciplinary team of providers. Existing research suggests providers are underprepared to assess and treat pediatric feeding disorder, therefore more information on training and clinical practice is needed. This study conducted focus groups to describe the training journey of providers across all four pediatric feeding disorder domains (medical, nutrition, feeding skill, psychosocial). Seven focus groups (total of 25 providers) were conducted and analyzed using thematic analysis. Four themes were identified: differences in academic preparation, workplace infrastructure and access, desire for comprehensiveness and feasibility, and value of the family perspective. Overall, results point to opportunities to improve provider training and therefore patient care including academic exposure to pediatric feeding disorder and multi-disciplinary collaboration practices, increased access to mentorship, training, and evidenced-based resources, and enrichment of the research to practice pipeline with a focus on family-centered care.

## Introduction

Pediatric feeding disorder (PFD) is oral intake that is not age-appropriate, lasts over two weeks, and is related to one or more of the following domains: medical, nutritional, feeding skill, and/or psychosocial [1]. Recent estimates suggest that the prevalence of PFD is up to 1 in 23 children under age 5 [2] with implications for children’s physical growth, cognition, emotional development, and caregiver relationships, as well as financial and emotional tolls on families [3–5]. PFD is heterogenous in presentation, affecting children across ages and requiring the care of a multidisciplinary suite of professionals in various settings. Appropriate and timely assessment and treatment of PFD is paramount in reducing the negative impacts and improving the health and well-being of children with PFD and their families, yet there is limited training for healthcare providers (e.g., speech language pathologists, psychologists, dietitians, occupational therapists), who report feeling unprepared to care for this population [6].

Research has demonstrated a lack of academic preparation related to feeding among healthcare professionals despite PFD being well within their scope of practice [6–10]. However, no clear direction has emerged about how to address this training gap. Further, little information exists about the training experiences of professionals throughout their career journey which may include achieving competency, expanding their skills, and/or providing training and mentorship. Preliminary research suggests speech-language pathologists and occupational therapists rely on expensive continuing education or self-directed mentorship to achieve competency in PFD, as well as to continue to grow their skills [11,12]. To our knowledge, there also is no formal education or training track or data regarding the need for training of psychosocial providers. Research that investigates not only the academic experiences but also the ongoing training of professionals across the PFD domains is needed to prioritize efforts to create a competent workforce to assess and treat this growing population of children.

### Aims and research questions

The purpose of the present study was to describe the training journey of professionals across all four PFD domains (medical, nutrition, feeding skill, psychosocial) to identify strengths, gaps, and needs in training. Focus groups with providers working across all four PFD domains were conducted to address the following aims:

1. Explore provider academic training experiences, including identifying formative experiences and opportunities for productive academic training experiences.
2. Understand provider training needs related to assessment tools and practices.
3. Explain provider experiences finding, evaluating, and implementing training in evidence-based treatments.
4. Describe provider experiences of evaluating clinical effectiveness and actions based on evaluation.

## Methods

### Design

Focus groups were chosen as they allowed for multidisciplinary discourse around the target subjects. Focus groups have been used in a wide range of social science inquiries and are beneficial for research that aims to highlight individual and group social dynamics [13]. This is aligned with the understanding that a team approach is integral to the assessment and treatment of PFD, including involvement across all four PFD domains [14]. This focus group study was approved the University of North Carolina at Chapel Hill Institutional Review Board IRB# 22–2515. All participants completed an online written consent form. Participants were additionally reminded of their consent and given an opportunity to rescind their consent at the start of the focus group.

### Sample and setting

This study recruited a multidisciplinary group of providers who assess and treat PFD from January 10, 2023 to March 13, 2023. We initially recruited a purposeful sample with maximum variation sampling to capture a diverse range of disciplines and work settings. Participants were recruited from a prior research survey [15] of professionals across domains about their clinical feeding practices where they had indicated they were interested in participating in further research on the topic, Feeding Matters email listservs, and emails to known contacts from specific disciplines and/or settings as needed. Feeding Matters is a national nonprofit system change organization headquartered in Arizona, dedicated to driving awareness, early identification, and comprehensive care for children with PFD and ARFID. Their work bridges the gap between families, healthcare providers, and institutions to create sustainable, systemic change that improves outcomes for children struggling with feeding challenges. They collaborate with families, medical professionals, and community champions to ensure children receive the care they need to thrive.

Interested professionals were directed to a Qualtrics survey that assessed eligibility and presented basic demographic questions. Inclusion criteria included experience assessing and/or treating PFD and availability to participate in a focus group. Participants who served on boards and committees related to PFD were excluded, as an intentional effort to allow for typical providers to provide their perspectives, achieving the goal of better understanding the provider landscape of PFD. We divided eligible participants into focus groups based on their discipline and practice setting, and then in order of response, in an effort to achieve balanced groups. Assigned participants were then emailed a copy of the consent form and a Qualtrics link to complete the consent and additional demographic information. Participants received a $25 gift card as a thank you for their participation.

### Data collection

A total of seven focus groups were conducted from January 2023 to March 2023 on Zoom. The groups were led by a researcher who was not familiar to the participants. Two Feeding Matters representatives also joined to take notes and monitor the chat but were muted and did not have their cameras on. Each session was audio and video recorded via Zoom with the participants’ consent.

A semi-structured focus group guide directed the focus group discussion. A multidisciplinary research taskforce including healthcare providers and researchers, developed a semi-structured focus group guide based on questions that emerged from the initial survey [15]. The focus group guide was piloted with two providers, an otolaryngologist and a gastroenterologist, and edited. Finally, the Feeding Matters Family Advisory Council reviewed the focus group guide; no additional changes were made based on their feedback.

Through a process of constant comparison, saturation of data from the medical and feeding skill domains was reached after four groups. Then, two groups of providers from the nutritional and psychosocial domains of PFD were completed to achieve saturation for those domains. After reflection, the NICU setting was not present in the existing groups, so one final group of providers in the NICU setting was conducted before complete saturation was reached.

### Data analysis

Initial transcripts were generated through the Zoom transcription service. Then, two master’s level speech-language pathology students checked and deidentified the transcripts. We loaded checked and deidentified transcripts into Atlas.Ti for coding and thematic analysis [16]. A thematic approach was chosen to qualitatively describe provider experiences across four topic areas by developing themes that surfaced across disciplines [17]. A combination of inductive and deductive coding was used. Under each topic area, a set of deductive codes were used to organize responses. One researcher (KT) coded all focus groups for the deductive codes. Next, two other members of the research team (CR and KF) reviewed these codes and responses with the goal of generating inductive codes. All focus group responses were coded using the collaborative set of inductive codes. In addition, this process yielded a combination of some deductive codes.

Once transcripts were coded, the team met to begin an iterative process of theme generation. Initial themes were organized by topic and then through discussion and transcript review, broadened to represent larger themes across topics. As part of this process the team took and compared notes, wrote and combined definitions of potential themes, and revisited original transcripts as well as codes. This process ultimately yielded four codes.

### Qualitative rigor

Researcher reflexivity is an important part of the research process. To achieve this, the primary team (KT, CR, and KF) met regularly throughout the research process to discuss immediate impressions post-group as well as regularly throughout the analysis process, including to reflect on potential biases. Qualitative rigor was achieved by targeting credibility, transferability, dependability, and confirmability [18]. Credibility was achieved using triangulation and member checks. Transferability was achieved through thick description of research participants and purposeful sampling. Using a team of researchers helped target dependability. Confirmability was addressed through researcher reflexibility as described above. Finally, the focus group leader (KT) was not involved in the original study development and therefore did not enter the focus groups with preconceived notions about responses. The focus group leader was also unknown to the participants which supported participant openness to providing honest feedback.

## Results

A total of 25 participants from multiple disciplines (physicians, speech language pathologists, occupational therapists, registered dietitians, nurse practitioner, board certified behavior analyst, psychologist) and practice settings were included in the seven focus groups (see Table 1). Focus groups ranged from 53–60 minutes. Four themes emerged from the analysis (see Tables 2 and 3).

**Table 1:** Participant characteristics.

		Overall (*n* = 24)	Focus Group						
			1 (*n* = 4)	2 (*n* = 5)	3 (*n* = 4)	4^a^ (*n* = 5)	5 (*n* = 2)	6 (*n* = 2)	7 (*n* = 2)
**Discipline**									
	BCBA	1	1	0	0	0	0	0	0
	NP	1	0	0	0	1^b^	0	0	0
	Physician	2	1	1	0	0	0	0	0
	OT	6	1	2	1	1	0	0	1
	Psychologist	4	1	0	1	1	0	1	0
	RD	4	0	1	1	0	1	1	0
	Social Worker	1	0	0	0	0	1	0	0
	SLP	6	0	1	1	3	0	0	1
**Work Setting**									
	Early Intervention/Home Health	5	0	2	0	2	1	0	0
	NICU	3	0	1	0	0	0	0	2
	Hospital Inpatient	2	0	0	1	1	0	0	0
	Outpatient Hospital/Rehab	4	0	1	1	2	0	0	0
	Multidisciplinary feeding clinic	7	2	0	2	1	1	1	0
	Private Practice	4	2	1	0	0	0	1	0
**Years of Practice**									
	10 or fewer	14	1	3	3	4	1	1	1
	11 or greater	10	3	2	1	1	1	1	1
**Gender Identity**									
	Male	3	2	1	0	0	0	0	0
	Female	21	2	4	4	5	2	2	2
**Race**									
	Asian or Asian American	1	0	1	0	0	0	0	0
	Black or African American	2	0	1	0	1	0	0	0
	Two or more races	1	0	1	0	0	0	0	0
	White	20	4	2	4	4	2	2	2

a One participant in this group did not report years of experience

b The nurse practitioner was also an international board certified lactation counselor

Notes. BCBA: board certified behavior analyst; NICU: neonatal intensive care unit; NP: nurse practitioner; OT: occupational therapist; RD: registered dietitian; SLP: speech-language pathologist

**Table 2:** Study aims to themes.

Theme	Study Aim
**Differences in Academic Preparation**	1. Training Experiences
**Workplace Infrastructure and Access**	1. Training Experiences
	3. Intervention
	4. Goals and Effectiveness
**Desire for Comprehensiveness and Feasibility**	1. Training Experiences
	2. Assessment
**Value of the Family Perspective**	2. Assessment
	3. Intervention
	4. Goals and Effectiveness

**Table 3:** Theme summaries.

Theme	Summary
Differences in Academic Preparation	Providers across disciplines reported feeling unprepared to effectively address PFD upon graduation from their academic institutions, regardless of the intensity of academic training. However, classes taught by clinicians or clinical exposure to PFD during their academic program directed providers to pursue PFD-specific jobs and training after graduation. Academic and clinical experiences differed by discipline.
Workplace Infrastructure	Providers reported different training, intervention, and both provider and client evaluation practices depending on their practice setting and discipline. Providers on multidisciplinary teams reported positive experiences with mentorship and robust resources for accessing, evaluating, and implementing evidence-based treatments. In contrast, providers in more siloed settings, such as private practice, reported training through continuing education and self-directed learning and the use of trial and error in applying intervention practices.
Desire for Comprehensiveness and Feasibility	Providers expressed a conflict between their ideal scenarios and those that would be realistic and feasible. This was exemplified by an overall desire for comprehensiveness and a simultaneous need for solutions that were financially and temporally feasible.
Value of the Family Perspective	Providers felt integration of family goals and perspectives were important across the care continuum. In particular, providers desired integration of caregiver goals into assessment and treatment plans and used report of caregiver satisfaction to evaluate the overall effectiveness of their treatment.

### Differences in academic preparation

Academic experiences, and their related clinical components, varied across disciplines. Participants who were speech-language pathologists (SLPs) or occupational therapists (OTs) tended to report having some coverage of feeding and swallowing during their academic training, although pediatric coverage was more limited. For example, one SLP said, “As far as pediatric and NICU [neonatal intensive care unit] feeding, I did not get any in graduate school we had the quintessential, “Read the peds chapter and we'll talk about it [in] lecture.’” In contrast, registered dietitians (RDs) and physicians were more likely to report never coming across PFD in their academic training. As one RD said, “I had minimal training in the area of pediatric nutrition. I think that's pretty common in the field still.” Psychologists reported training in PFD during their doctoral clinical fellowships, as described by one participant, “I did my pre doctoral internship in a multidisciplinary day treatment feeding program. Did my postdoc at a multi-disciplinary feeding program.”

Regardless of academic experience, across disciplines, providers reported feeling unprepared to effectively address PFD following graduation from their academic institutions. This was true regardless of their academic training. As one participant said, “Coming out of grad school, I had a little bit of a grasp on feeding disorders and feeding therapy, and just feeding in general, but I didn't feel very confident in it.” While feelings of unpreparedness were a universally reported phenomenon, some forms of academic preparation were effective in igniting interest in PFD and directing providers to pursue post-graduate careers or training related to PFD. For example, classes related to PFD that were taught by practicing providers were impactful, as put by one participant, “I have a unique experience… my dysphagia course in grad school was taught by someone from the local children's hospital instead of someone who was adult focused … And then … a birth to 3 class, and it was taught by somebody who did the NICU.” Additionally, clinical exposure to PFD during the academic program, such as through clinical placement or observations, was effective. One participant put it this way, “I started in the outpatient context and did a lot of observations with other therapists, and just really grew to love working with children with medical complexities.”

### Workplace infrastructure

Practice setting significantly impacted provider-reported training, intervention, and evaluation practices. In terms of training, providers who were members of multidisciplinary teams reported positive experiences with mentorship. As one participant said, “The most efficient, effective training was just working with my team members and working in the clinic...just really learning and training from the other disciplines in our team was very valuable.” This mentorship was successful in achieving well-rounded clinical knowledge across disciplines as stated by another participant, “I got the most additional education from my coworkers. Because when I began working at a very established feeding program...they had many years of experience working with different types of pediatric feeding disorders.” In contrast, providers who were not members of PFD-specific teams (e.g., early intervention, private practice, home health) sought training in different ways. Specifically, these providers mentioned seeking out continuing education events. One participant described this, “For myself, it was because I had no choice. The kids added up on my caseload, and I had to learn. So I went in and started training for all the kids that were dropping in...” These providers reported needing to be more self-directed in their learning because they did not have structured opportunities to gain additional skills without seeking it out themselves.

When it came to providing evidence-based treatment, providers on multidisciplinary teams reported having access to robust resources for doing so. Access to peer-reviewed research was one facilitator of providing such intervention, as described by one provider, “I feel very lucky too that we're within a large institution where I do have easy access to a lot of peer-reviewed journals. So, I can go to the research and access evidence-based recommendations that I can share with families.” In addition, providers on teams benefited from learning from other team members who were abreast of different research. Teams that were more medically integrated (e.g., NICU) also had additional resources, such as nurses and fellows who were engaged in research and could bring this to the team. A NICU provider explained, “One way that [EBT] comes in is that since we're part of the NICU Research Network, one of our clinicians is … a faculty member and she actually works on research projects with the neonatology fellows.” A further facilitator of evidence-based treatment on multidisciplinary teams was the use of practice guidelines which outlined evidence-based treatment options for different PFD presentations, as mentioned by one provider, “At my institution, we have what we call clinical care guidelines ... [which] depict a pathway for each type of patient.” Providers who worked individually did not often have access to these resources. Instead, they used continuing education events coupled with self-reported ‘trial and error’ to provide evidence-based, individualized treatment to their clients. This was described by one provider like this, “There's often a lot of you know, trial and error, especially early on, and working with the family, getting that rapport going. Let's try this, and then you, as a provider … looking at how the intervention is improving function or not, or how we can kind of keep working with the family and tweaking [approaches]” and another like this, “A lot of times I get the education, and I read through the research articles, and I go, hey? Why not? Let's try it, and you know, maybe it works. Maybe it doesn't. That's gonna be kind of my evidence. Obviously, as long as it's not a safety hazard for whoever I'm working with. But I feel like you know I can try it if it works for me, great. If it doesn't work, then I've tried something, and I know I can try something else next.”

Finally, there were some differences in provider evaluation practices based on practice setting. Members of multidisciplinary teams reported more structured and formal self-evaluation processes. For example, patient satisfaction surveys were automatically sent by the larger institution providers worked for and helped them evaluate their effectiveness as clinicians. They also reported other formal avenues for peer feedback such as, “I do have monthly reflective supervision with my supervisor, being able to take the time to think about tricky cases, what could we have done differently? Did we have a misstep? Was there something we could have done to engage them better? What can we do next time?” Providers who worked individually self-evaluated in more qualitative ways. For example, family satisfaction was an important metric, and was evaluated based on the rapport, relationship, and direct feedback providers gained from families. This was described by several providers such as, “I mean, it's not objective at all, but when you're clicking the parents, or you're not [or] when you make a difference versus not.” Another provider described it this way, “As far as whether I'm successful or not, a lot of it depends on whether my parents are happy with the child. Do they feel successful? Do they feel like this child is now able to participate in the family meal?” While providers from both settings mentioned self-reflection as a method of evaluating clinical effectiveness, providers practicing individually tended to report this more often as they did not have the opportunity for direct feedback from team members.

### Desire for comprehensiveness and feasibility

When discussing training and assessment, providers expressed a conflict between their ideal scenarios and those that would be realistic and feasible. For example, the vast majority of providers mentioned a desire for more comprehensive academic training, as one participant stated, “I wish that I had gotten more information about the different approaches to feeding therapy [in school], because I feel like that's a point of discussion daily with parents, with other coworkers, and with other professionals.” However, providers also acknowledged the breadth of information that was expected to be covered by their academic programs. One participant put it this way, “I know there's a very limited amount of time and a lot of stuff that they have to cover. And I get that. But it was it was kind of scary coming out of grad school and being expected to just come pick up with these feeding kiddos that were complex and a little bit scary.” Therefore, while providers desired comprehensive academic training, they also acknowledged this may not be feasible.

Providers also desired more comprehensive training after graduation but acknowledged feasibility limitations to accessing such training such as being able to take time off of work to attend and the cost of training. This conflict led providers to desire alternative solutions, as described by one participant like this, “You do need that mentorship [when you finish school] and that extra training and being in a rural area, there's definitely a significant cost to obtaining that. And I think that [cost] is a barrier for some folks kind of pursuing this area.”

Finally, competing desires for comprehensiveness and feasibility were exemplified when discussing an ideal or gold standard assessment. Providers across disciplines described an ideal assessment that covered all four domains of PFD comprehensively in order to capture the nuances of PFD. This was described by one participant like this, “[The ideal assessment would have] the elements that affect PFD such as kind of the medical piece, and nutrition piece, oral motor skills, sensory, behavioral and those elements.” However, providers also emphasized that the ideal assessment would also be easy to complete and able to be completed in a short time window. For example, one participant described their ideal assessment as needing to, “be concise and effective for the family, but also the provider.” Providers desired a universal assessment to support efficient and effective assessment of children with PFD while describing this tool as being unavailable.

### Value of the family perspective

Across focus groups, providers emphasized the importance and value of including the family across the care continuum. When it came to assessment, providers specifically mentioned the importance of integrating the family perspective, experience, and goals into a gold standard assessment. For example, one participant shared, “I also think it would be important to have a parent perspective component or parent expectation, because they're the ones really doing a lot of the work.” In fact, several providers felt the family perspective was missing from currently available assessments, as described by one participant, “When you're talking about a new infant and a new parent, so much of that child's outcomes relate to how confident the parent feels in being able to care for their baby and advocate for their baby. And most standardized assessments are very clinical and completely leave out that one piece that has the most impact.” Similarly, when it came to intervention, many providers prioritized and incorporated family goals into their treatment approach. For example, one provider explained, “I also have changed in the last couple of years of really asking what family's goals are, and writing really simple goals like, if a family really wants their baby to touch and explore a smash cake at their first birthday, I'm like, alright, we're gonna put that as a goal.”

In addition to assessment and treatment, many providers highly valued family feedback when evaluating the effectiveness of their treatment. These providers tended to be SLPs or OTs. There were several family-centered evaluation practices described. Most common was informally integrating family feedback. For example, “I try and get kind of a mix of both [types of evaluation], the more like subjective, how is it going from the family's perspective, as well as something objective.” and “We mainly do assess what their [the child’s] treatment goals [are], what the family's treatment goals are, maybe what our goals are”. Family satisfaction with services was also important in clinicians’ report of their own self-evaluation of effectiveness. Less commonly reported were more formal measures of family feedback, such as formal family review forms.

## Discussion

The present study explored provider experiences of PFD training to identify opportunities for advanced training across disciplines. Identified opportunities can inform the PFD community of provider education needs in order to strengthen training pathways and workforce development. Four themes were identified: 1) differences in academic preparation, 2) impact of infrastructure, 3) desire for comprehensiveness and feasibility, and 4) value of the family perspective. These themes enhance understanding of the broader context of PFD training and provide actionable avenues for strengthening provider preparedness across disciplines and settings. The findings of this study can direct future educational opportunities across domains thereby improving the system of care for children with PFD and their families.

### Academic experiences and opportunities

Academic experiences set the stage for providers’ later clinical and training experiences. When conceptualizing these provider experiences, it is important to remember that academic programs vary significantly across and within the many disciplines who work with children with PFD. This includes differences in entry level degree type – Masters for Board Certified Behavioral Analyst (BCBA), SLPs, and OTs; Medical Doctorate or Doctor of Osteopathic Medicine for physicians, and others – and differing coverage of PFD during this academic training, even within disciplines [11]. Findings from the present study can provide future directions for how to increase provider confidence and competence upon graduation. For example, participants in the present study who were given foundational or introductory information on PFD during their academic studies reportedly knew how to access PFD training early on in their careers, consistent with quantitative findings from a study about SLPs and OTs academic and post-graduate PFD training experiences [11]. They also reported feeling better supported in their early careers, regardless of the degree to which their academic program delved into PFD assessment and treatment. Therefore, while in-depth coverage of PFD in academic programs would be preferred to evenly prepare providers across a range of settings, a minimum requirement of exposure to PFD during academic programs could lead to better early support, as providers would be better equipped to know how and where to effectively seek out additional training regardless of practice setting. This also addresses provider knowledge that while advanced PFD training during their academic experiences would be ideal, their academic programs have a great deal of information to cover [19]. Other findings that provide direction for future academic program development include the endorsement of clinician-led classes on PFD and the value of clinical observation during academic training which are aligned with current research on the scholarship of teaching and learning [20,21]. One possible avenue for exposure to PFD during academic training could be to complete a curricular needs assessment [22] and create a set of core areas for professional activity [23] to guide academic training; a model set out by medical schools to prepare residents to care for children with medical complexity. Methods for education include a combination of simulation-based education, clinical rotations, traditional didactic education, and/or family-centered education [24–28]. For example, while limited in number, some Master’s level BCBA programs offer a training model where academic and clinical preparation is fully integrated into a multidisciplinary feeding team, providing students with early immersive and specialized training.

### Ongoing training

Regardless of academic training, all clinicians endorsed a need for continual professional development around PFD. Pathways for achieving continuing education related to PFD varied, partly based on place of employment. The differing level of opportunities based on practice setting represents a potential inequity in PFD-related practice that should be addressed by future research and advocacy work.(11,12) One avenue for addressing these inequities is to expand access to quality mentorship across disciplines in both settings. Connecting providers with advanced training to those earlier on their journey working with PFD could lead to better support for those who may otherwise not have access to early career guidance [29] and is supported by research investigating the desires and needs of providers when it comes to PFD [6]. Additionally, academic medical institutions could support local community providers through innovative practices such as sharing access to grand rounds, offering community education seminars and supporting smooth care transitions through collaborative teaming and education. Finally, clinicians in the focus groups emphasized the importance of cross-disciplinary training. However, service delivery settings, disciplinary roles, and billing structures impact the degree to which this can occur. For example, newer clinicians in some disciplines may have more independent billing “power” but less hands-on experience. This service delivery structure may limit the ability of experienced clinicians to provide mentorship outside of their discipline as well as impact the ability to gain competency as an independent provider. Addressing these challenges would require broad advocacy efforts across disciplines.

### Training components

The present study asked providers to report on current assessment and treatment practices and needs to assess gaps and opportunities for training.

### Assessment

Providers in the present study were united in their desire for assessments that address all four domains of PFD in a way that is feasible given the time constraints of their respective practice settings, as is current best practice [30]. This includes in-depth assessment within the provider’s own domain and screening and referral guidelines for other domains. However, there are the limited tools available that address all four PFD domains in a systematic way [31]. While these kinds of tools are being developed, preparing clinicians to accurately screen and assess PFD necessitates cross-disciplinary training both at the academic and post-graduate levels. Without such training, medical complications, feeding skill deficits, nutritional risk, and psychosocial dysfunction may not be identified. Late or lack of identification can result in long-term disability in children with PFD [1].

In addition, numerous providers in the present study emphasized the value of incorporating family perspectives during assessment to guide clinical decision-making which mirrored findings by Rabaey and colleagues [31] who found providers desired a connection to the family impact of PFD. This demonstrates a consistent underlying ideology that recognizes the impact of PFD on the entire family, not just the individual child [32,33]. Training on assessment of PFD should incorporate how to integrate the family perspective using valid and reliable tools such as the patient reported outcome measures reviewed by Marshall et al. [34] (e.g., Feeding Impact Scales) [35]. Research should continue to establish sound psychometric properties for other tools, such as the Parenting Stress Index, the Family Management Measure of Feeding, and the Behavioral Pediatric Feeding Assessment Scale (for populations outside of autism) [36–38].

### Treatment

All participating providers in the present study emphasized the importance of evidence-based practice. It is critical that all professionals are prepared during their academic programs to find and critically evaluate research evidence [39]. This academic training will prepare providers to critically assess the integrity of training on treatment that is presented to them and its suitability for their patient. Methods for academic training on research evaluation have included workshops, toolkits/guidelines, online support, and opportunities to practice [40]. Additionally, efforts should be made to neutralize disparities between providers in community versus medical institution settings when it comes to access to research evidence. Granting organizations and research institutions are encouraged to rethink their dissemination requirements to decrease inequities perpetuated by current institutional policies about journal access [41]. Specific actions could include encouraging researchers to communicate with providers through open access publishing and allotments to broadly share findings through conferences and social media.

Similarly to assessment, providers also mentioned the importance of the family perspective when it came to choosing treatments, further indicating a desire from providers for family-centered care to be a core element of training. The importance of family-centered approaches have been reinforced in the literature, with evidence they have the potential to improve the quality of life of children with PFD [33], and that caregivers of children with PFD have indicated a preference for healthcare providers who prioritize a family-centered approach [3]. Balancing family-centered care with evidence-based practice currently falls on the provider and their training model. For example, some providers may have broader experiences in post-graduate training (e.g., attending multiple different continuing education events, mentorship from experienced clinicians) which may contribute to flexibility around shaping treatment to the child and family’s needs. Future research that is designed to investigate clinical reasoning based on family goals, verses predetermined or medically directed protocols, could support the use of family-centered care across settings. Additionally, this kind of research could give community providers the opportunity to share their expertise in flexibility and clinical decision making.

### Evaluation and reflection

Evaluation of provider effectiveness is a key element of the training journey. Clinical practice is cyclical – we assess and treat, reflect on what has gone well and where we could improve, and then seek training to build our skills. Encouragingly, providers in the present study reported participating in this process. Participants used methods such as self-evaluation and reflection, as well as more formal means like performance reviews and checklists. Family feedback was also a key component of provider evaluation across disciplines and settings. Some family feedback came in more formal forms (e.g., patient satisfaction surveys, referrals to the provider) while others were more personal (e.g., a family inviting the clinician to their child’s birthday party, parents expressing gratitude to provider). Research demonstrates that timely formal feedback, as well as family feedback in the form of patient reported outcome measures, can improve aspects of patient care and healthcare provider satisfaction, especially when paired with practical strategies like checklists and tools [42–45].

### Limitations

The present study offers a novel exploration of provider perspectives on PFD training journeys and there are several limitations that should be noted. The sample in the present study was largely recruited from members of the Feeding Matters network who had consented to engaging in research, which could have been a barrier to accessing a sufficiently diverse pool of participants. Specifically, Feeding Matters tends to attract providers who are more heavily involved in PFD related training and experiences. A multidisciplinary structure was chosen for the focus groups to reflect the multidomain nature of PFD, but this structure may have hindered more in-depth perspective sharing from some members due to the hierarchical nature of healthcare delivery. There are benefits to using virtual platforms such as expanding access for participants, cost and time efficiency, and the ability to recruit a national sample; although participant engagement can be reduced in this setting [46]. Future studies could maximize the utility of the virtual space by integrating features such as screen polling or breakout rooms. Finally, while this study focused specifically on PFD, many PFD professionals also work with children and adults with avoidant restrictive food intake disorder (ARFID). Understanding professional training and preparation for ARFID and PFD can assist in better preparing clinicians for feeding related work more broadly. Therefore, future work should examine the preparation of professionals across disciplines related to ARFID.

## Conclusion

This study found that training related to PFD is an ongoing journey that begins with initial exposure in the academic setting and continues as the provider assesses, treats, and reflects on their effectiveness. Opportunities to improve the educational system related to PFD emerged. These opportunities include exposure to PFD across disciplines during academic training (to include awareness of the PFD diagnostic criteria and screening practices to determine domain involvement/expression), exposure to multi-disciplinary collaboration practices and scenarios in academic settings, increased access to mentorship, training, and evidenced-based resources regardless of academic affiliation, and enhancement of the research to practice pipeline with a focus on family-centered care. Addressing provider needs across practice settings and domains can lead to a more consistent, holistic, and productive treatment experience for children with PFD.
